# Cost-Effective Cosmetic-Grade Hyaluronan Hydrogels for ReNcell VM Human Neural Stem Cell Culture

**DOI:** 10.3390/biom9100515

**Published:** 2019-09-20

**Authors:** Weili Ma, Won Hyuk Suh

**Affiliations:** Department of Bioengineering, College of Engineering, Temple University, 1947 N. 12th St. Philadelphia, PA 19122, USA; weili.ma@temple.edu

**Keywords:** ReNcell, human neural stem cell, methacrylated hyaluronic acid, hydrogel, differentiation

## Abstract

Hyaluronic acid (HA) is a polysaccharide polymer frequently used as a starting material to fabricate hydrogels, especially for recapitulating the brain’s extracellular matrix (ECM) for in vitro neural stem cell (NSC) cultures. Here, we report the successful synthesis of a methacrylated HA (MeHA) polymer from an inexpensive cosmetic-grade hyaluronan starting material. The MeHA polymers synthesized from cosmetic-grade HA yielded similar chemical purity to those from pharmaceutical/research-grade HA reported in the literature. Crosslinked MeHA (x-MeHA) hydrogels were formed using radical polymerization which resulted in mechanical properties matching previously reported mechanical property ranges for enhanced neuronal differentiation of NSCs. We assessed cellular adhesion, spreading, proliferation, and stiffness-dependent neuronal differentiation properties of ReNcell VM human neural stem cells (hNSCs) and compared our results to studies reported in the literature (that utilized non-human and human pluripotent cell-derived NSCs).

## 1. Introduction

Hyaluronic acid (HA) is frequently utilized as the base material for fabricating synthetic substrates that promote cell adhesion due to its structural role in the native brain’s extracellular matrix (ECM) [1]. HA-based hydrogels, for this reason, have been widely investigated as a key component in engineered microenvironment studies involving neural stem cell (NSC) differentiation processes [2,3,4,5,6,7]. The HA hydrogel systems reported in the literature, however, exclusively use pharmaceutical-or-research-grade HA, making large-scale production a cost ineffective endeavor. We sought to test the biocompatibility of cosmetic-grade HA, to circumvent the cost issues, which is already FDA approved for applications such as topical creams and dermal fillers [8]. These cosmetic HA powders can be obtained at significantly lower costs (by approximately 100-fold), providing the potential to use HA-hydrogels in high-throughput but cost-effective studies. Among the different chemistries available for HA modification and crosslinking [9], we chose to introduce the methacrylate functionality to the HA backbone for radical polymerization capabilities [10]. Compared to other types of chemical crosslinking, radical polymerization of methacrylated HA (MeHA) using light energy (i.e., UV-based photopolymerization) occurs at a rapid rate (e.g., 1–10 min) with excellent spatio-temporal control over the reaction [11]. Furthermore, crosslinked MeHA (x-MeHA) hydrogels fabricated using this method have also been reported to have good biocompatibility with neuronal cell types [2,6].

Independent variables (also known as cues) that control stem cell differentiation have been of interest for thorough investigation due to their potential utilization as modulation points for the improvement of clinical outcomes [12]. One specific example is the induction of neuronal cells from neural stem cells to treat neurodegenerative diseases [13,14]. Aside from chemical stimulation strategies, mesenchymal stem cells (MSCs) were reported to undergo lineage-specific differentiation based on the stiffness of their surrounding environment [15]. Following this pioneering study, correlation studies involving stiffness properties and differentiation have been reported in other adult stem cell types, including NSCs [16,17]. It is now generally accepted that NSCs prefer neuronal differentiation on softer substrates (<500 Pa) [16,17,18,19,20]. While the results from these early works were with non-human NSCs, recently published studies have begun to report correlation results involving the stiffness versus differentiation of human NSCs (hNSCs) derived from embryonic stem cells (ESCs) [7] and induced pluripotent stem cells (iPSCs) [6]. There are, however, several key limitations in using ESC-and-iPSC-derived hNSCs. For ESCs, the problem is with bioethics, since the method of cell isolation results in the destruction of an embryo [21]. While iPSCs do not have the same ethical dilemma, they are known to have epigenetic memory which can influence their differentiation fate [22]. For this study, we used ReNcell VM, an hNSC line that was immortalized by v-myc transfection [23]. This cell line has been used in previous studies to model human disease [24] and study chemical differentiation pathways [25] and methods [26].

## 2. Materials and Methods

Hylauronic Acid (Low Molecular Weight, 8.5 kDa MW average) was purchased from CosChemSupply (Rancho Cucamonga, CA, USA). Methacrylic Anhydride (760-93-0) was purchased from Alfa Aesar (Haverhill, MA, USA). Sodium Hydroxide (S5881), Deuterium Oxide (151882), Irgacure 2959 (410896), Donkey Serum (D9663), Laminin (CC095 and L2020), ReNcell VM (RVM, SCC008), ReNcell Maintenance Media (RMM, SCM005), Accutase (SCR005), Epidermal Growth Factor (EGF, GF144), EmbryoMax^®^ Dulbecco’s Phosphate Buffered Saline (PBS, BSS-1006-B), Dulbecco’s Modified Eagle’s Medium with Ham’s F12 Nutrient Mixture (DMEM/F12, DF-041-B), and FITC-Labeled Secondary Antibody (AP182F) were purchased from MilliporeSigma (Burlington, MA, USA). Penicillin-Streptomycin (Pen-Strep, 30-002-CI) was purchased from Corning (Corning, NY, USA). Paraformaldehyde (PFA, AC41678) was purchased from ThermoFisher Scientific (Waltham, MA, USA). Beta III Tubulin mouse monoclonal primary antibody (TU-20) was purchased from Cell Signaling Technology (Danvers, MA, USA). Basic Fibroblast Growth Factor (bFGF, 100-18B) was purchased from PeproTech (Rocky Hill, NJ, USA). Cell culture related (disposable) products such as plasticware (not mentioned above) were purchased from ThermoFisher Scientific (Waltham, MA, USA).

### 2.1. Synthesis of Methacrylated Hyaluronic Acid (MeHA)

The methacrylation of HA was carried out utilizing previously established protocols [2,10]. Low molecular weight hyaluronic acid was dissolved in deionized water at 1% *w*/*v* concentration by magnetic stirring. The solution was chilled on ice for 5 min and the pH was adjusted to 8.0 using 5 N NaOH. While stirring on ice, 20 mol. eq. (relative to primary hydroxy group on the HA *N*-acetylglucosamine subunit) methacrylic anhydride was added dropwise to the HA solution. The reaction was carried out for 2 h on ice and the pH was maintained at 8.0 by the continuous addition of 5 N NaOH. The reaction solution was transferred to a glass graduated cylinder and was left for 15 min at room temperature. The reaction solution separates into three layers: an aqueous top layer containing the polymer product, an interphase containing methacrylic acid byproduct, and a lower organic phase containing unreacted methacrylic anhydride. The aqueous layer was carefully removed and transferred to dialysis membranes (3.5 kDa pore size) and dialyzed against distilled water for 72 h. Dry product was obtained by lyophilization and was stored desiccated at −20 °C until use. The degree of methacrylation was calculated based on ^1^H-NMR spectral measurements [27]. Lyophilized MeHA was dissolved in deuterium oxide at 1% *w/v*. Spectra were obtained on a Bruker Avance III HD 500 MHz instrument. Degree of methacrylation was calculated using integrations based on three different methodologies previously reported in the literature [28,29,30].

### 2.2. Crosslinked Hydrogel Fabrication

A photoinitiator solution was prepared by dissolving Irgacure 2959 in deionized water at 0.1% *w*/*v*. Lyophilized MeHA was dissolved in this photoinitiator solution to a final polymer concentration of either 2.5% *w*/*v*, 5% *w*/*v*, or 10% *w*/*v*. Crosslinking was achieved by exposing the precursor solution to a 302 nm UV lamp (UVP, LLC; Part of Analytik Jena; Jena, Germany) for 5 min. For cell experiments, the precursor solutions were filtered through a 0.22 µm membrane prior to gelation in an aseptic environment. The hydrogels were rinsed three times with PBS, followed by a 30 min incubation at 37 °C with PBS. Afterwards, hydrogels were kept in proliferation media at 37 °C (formulation detailed in cell culture section below) until cell seeding. Laminin coatings on hydrogel surfaces were performed using the same protocol described below for the preparation of polystyrene surfaces for cell culture.

### 2.3. Rheology

The viscoelastic properties of the hydrogels were measured by oscillatory frequency sweeps on an ATS StressTech rheometer fitted with a parallel plate stage (ATS Rheosystems; State College, PA, USA). Hydrogels were freshly fabricated in a silicone mold with set dimensions (0.5 inch diameter and 2 mm thickness). The hydrogels were swollen in PBS for 30 min after fabrication. Measurements were taken over 0.1 to 10 Hz at a constant 0.5% strain. Hydrogels from multiple synthesis batches and combination of synthesis batches were tested.

### 2.4. Scanning Electron Microscopy (SEM)

SEM was used to visualize the morphology and the pore sizes of the crosslinked hydrogel network. After UV exposure, the crosslinked hydrogels were lyophilized and placed onto glass slides using double-sided carbon tape. After sputter coating, SEM micrographs were obtained on an Agilent 8500 FE instrument. The images were imported into ImageJ (v1.50i or later, National Institute of Health, USA) [31,32] for manual measurements of pore diameters.

### 2.5. Stem Cell Culture

ReNcell VM were cultured on laminin-coated polystyrene flasks (ThermoFisher BioLite, 25 cm^2^ or 75 cm^2^ growth area). To prepare the laminin-coating, laminin was diluted to 20 µg/mL in DMEM/F12 and incubated at 37 °C for 4 h with polystyrene surfaces or hydrogels. Cells were kept in proliferation with ReNcell maintenance medium supplemented with 20 ng/mL EGF, 20 ng/mL bFGF, and 100 µg/mL Pen-Strep. Cell medium was refreshed every 48 h during proliferation. Differentiation medium was prepared by not incorporating growth factors into the media [23]. For hydrogel experiments, 50 µL of hydrogel precursor aqueous solution was aliquoted into each well of a 96-well plate. Any air bubbles were removed with a sterile pipette tip prior to UV crosslinking. The hydrogels were rinsed three times with sterile PBS and then incubated for 30 min with proliferation media prior to cell seeding. The proliferation media was removed and refreshed at the time of seeding.

### 2.6. Analysis of Cell Adhesion and Spreading

Hydrogels were prepared for cell culture (described in the hydrogel fabrication section above) in 96-well plates. In the same plate, wells were laminin-coated for control conditions and standard curve generation. Cells were detached by 5 min treatment with Accutase) at room temperature (or 37 °C) followed by pelletization by centrifugation at 200× *g*. After cell counting (in the presence of trypan blue), 1 × 10^4^ cells were seeded into each well. The total proliferation medium volume per each well was 100 µL. The samples were incubated at 37 °C for 1 h, at which point cell adhesion and spreading were confirmed by microscopy. After removing the media, each well was rinsed carefully three times with PBS. For cell adhesion measurements, PrestoBlue was diluted 1:10 in media to make the working solution, and 100 µL was incubated with the samples at 37 °C for 1 h. Afterwards, 50 µL was transferred to new 96-well plates for fluorescence measurements (560/590 nm ex/em) on a Tecan Infinite M200 Pro plate reader (Tecan Group, Männedorf, Switzerland). After background subtraction with samples without cells (i.e., only PrestoBlue solution or PrestoBlue solution with hydrogel), cell adhesion to the hydrogel surface was normalized to total cell adhesion on the laminin-coated wells. For cell spreading analysis, cells were incubated under the same conditions with 2 mM Calcein AM instead of PrestoBlue. Live images of cells on the laminin-coated wells or hydrogel surfaces were obtained using an Olympus IX83 inverted microscope fitted with an LCI Chamlide live-cell stage-top incubator system. These images were imported into ImageJ for circularity analysis, which was used as a determinant of defining the degree of spreading [33].

### 2.7. Proliferation and Differentiation

1 × 10^4^ cells were seeded onto laminin-coated wells or hydrogels with laminin. For 72 h, cell proliferation was monitored using live-cell microscopy. Media were refreshed daily during this process. After 72 h, differentiation was initiated by growth factor withdraw and media were refreshed every 48–72 h for 1 week. At this point, samples were fixed by with 4% PFA (10 min incubation). After removing the PFA solution, cells were kept at 4 °C in PBS until antibody staining. The following immunocytochemistry protocol was performed at room temperature unless otherwise stated. After rinsing three times with PBS, samples were blocked and permeabilized by treating for 30 min with a 0.22 µm filtered solution of 5% *v*/*v* donkey serum and 0.3% Triton X-100 in PBS. Primary antibody for the neuronal marker βIII-tubulin (Cell Signaling Technology TU-20, Danvers, MA, USA) was diluted 1:200 in the aforementioned blocking and permeabilization solution. The samples were incubated with primary antibodies overnight (16–24 h) at 4 °C. Afterwards, samples were rinsed three times with PBS. Secondary fluorophore-labeled antibody (MilliporeSigma AP192F, Burlington, MA, USA) was diluted 1:500 in the blocking and permeabilization solution. Secondary antibodies were incubated with the samples for 1 h in the dark. Nuclei were stained by incubating for 15 min in the dark with Hoechst 33342 (ThermoFisher H3572, Waltham, MA, USA) diluted to 10 µg/mL in PBS. Samples were rinsed three times with PBS and were kept hydrated in PBS during imaging on an Olympus IX83 inverted microscope. Z-stack images of differentiated spheroids were obtained using a 1 or 2 µm slice thickness. The z-stacks were imported into ImageJ and a maximum projection over z was generated for each channel. The total area of the spheroids and area (pixels) of βIII-tubulin were measured for each image. The data was analyzed by taking the ratio of βIII-tubulin to total spheroid area, giving an estimate of percent βIII-tubulin per spheroid.

### 2.8. Statistical Analysis

All statistical analysis was performed on Microsoft Excel and JMP Pro (version 13 or 14, SAS Institute, Cary, NC, USA). Two-tailed Student’s *t*-tests were performed to compare rheological results. Cell adhesion was compared by two-tailed Student’s *t*-testing between each sample. Pore sizes of hydrogels and neurosphere sizes during proliferation were compared using a one-way ANOVA with post hoc two-tailed Student’s *t*-tests between each group. βIII-tubulin expression after differentiation was compared by a two-tailed Student’s *t*-test. A *p*-value less than 0.05 was considered statistically significant. Hydrogel testing results were pooled from, at least, three independent synthesis batches of MeHA. All biological experiments were performed with technical replicates at least three times, independently. Numerical data are presented in the text as mean ± standard deviation (S.D.) or standard error of the mean (S.E.M.) unless otherwise stated.

## 3. Results

### 3.1. Synthesis of Cosmetic-Grade MeHA and ^1^H-NMR Characterization

**Figure 1:**
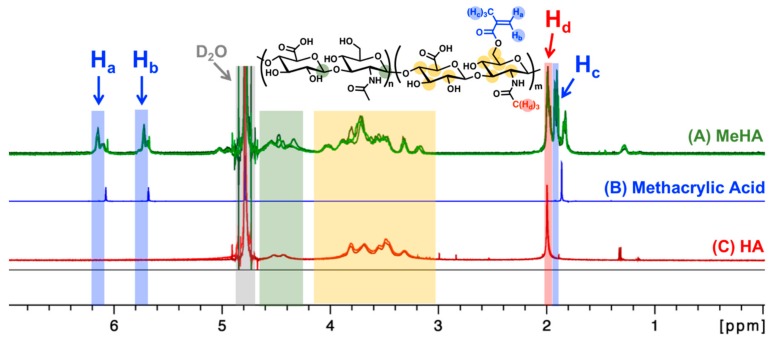
^1^H-NMR spectra of methacrylated hyaluronic acid (A, MeHA; three samples; green lines), methacrylic acid (B, blue line), and unmodified hyaluronic acid (C, HA; two samples; red lines). Methacrylate peaks (blue arrows; δ 6.2 ppm, δ 5.8 ppm, δ 1.9 ppm; H_a_, H_b_, H_c_) were used to calculate the degree of methacrylation (Table 1). The methyl group proton on the *N*-acetylglucosamine is between δ 2.0–2.1 ppm (red arrow; H_d_). D_2_O solvent peak was set to 4.79 ppm (gray area) [27]. The color-coded protons in the chemical structure (top center) are expected to show up in the color-matching boxed regions within the ^1^H-NMR spectra (A–C).

Methacrylation of HA was carried out using an oligomeric low molecular weight (8000–15,000 Daltons), cosmetic-grade HA. As seen in the ^1^H-NMR spectra presented in Figure 1 below, additional peaks corresponding to the methacrylate group are observed after the reaction (blue arrows), indicating successful conjugation of the methacrylate pendant group.

As discussed previously in the methodology, there are three reported methods to estimate the degree of methacrylation. In the first method [28], the integration is performed on the two protons of the methylene carbon on the methacrylate group at δ 6.2 ppm and δ 5.8 ppm in relation to the ten protons of the backbone structure between δ 3.0–4.2 ppm (yellow region in Figure 1). The second method [29] looks at the sum of the five methacrylate protons at δ 6.2 ppm, δ 5.8 ppm, and δ 1.9–2.0 ppm (blue regions in Figure 1) in relation to the three protons on the methyl groups of the *N*-acetylglucosamine subunit at δ 2.0–2.1 ppm (red region in Figure 1). Finally, the third method [30] calculates degree of methacrylation by comparing the two methylene protons of the methacrylate group at δ 6.2 ppm and δ 5.8 ppm to the two anomeric protons of the backbone structure between δ 4.2–4.8 ppm (green region in Figure 1). The estimated degrees of methacrylation, calculated using all three methods, are presented in Table 1. There were no statistical differences amongst the values acquired from the three different calculation methods and the mean methacrylation value came out to be approx. 57%.

### 3.2. MeHA Hydrogel Fabrication and Physical Characterization

**Figure 2:**
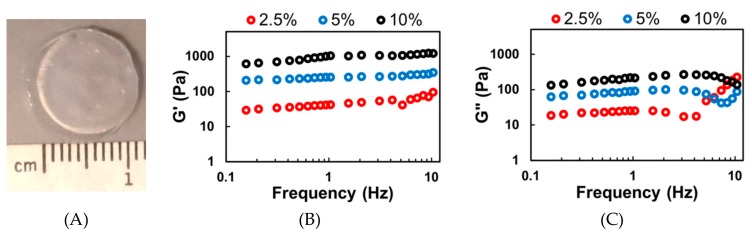
Rheological analysis of photopolymerized and crosslinked Me-HA (x-MeHA) hydrogels. (**A**) Photograph of a crosslinked x-MeHA hydrogel. Averaged (**B**) storage modulus (G’) and (**C**) loss modulus (G”) measurements from hydrogels fabricated using 2.5% (red), 5% (blue), or 10% (black) *w*/*v* MeHA. N ≥ 15 hydrogels per condition.

Hydrogels were successfully fabricated using a 302 nm UV light source and the Irgacure 2959 photoinitiator (Figure 2A). The concentration of Irgacure 2959 (0.1% *w*/*v*) and the UV light exposure time (5 min) were kept constant. By modulating the polymer concentration, hydrogels with varying viscoelastic stiffness could be fabricated. The storage (G’) and loss (G”) moduli are presented below, in Figure 2B,C. During static cell culture, the hydrogels should not experience high frequency shear stress. Thus, the storage (G’) and loss (G”) moduli (mean ± standard deviation) were calculated using the measured values between 0.1 to 1 Hz. Hydrogels fabricated using 2.5% *w*/*v* MeHA resulted in a G’ of 41.9 ± 25.2 Pa and a G” of 4.8 ± 3.4 Pa. Increasing the MeHA concentration to 5% *w*/*v* resulted in significantly stiffer hydrogels with a G’ of 265.1 ± 87.2 Pa and a G” of 20.2 ± 11.7 Pa. Further increasing to 10% *w*/*v* resulted in hydrogels with a G’ of 933.6 ± 169.1 Pa and a G” of 199.5 ± 39.2 Pa.

**Figure 3:**
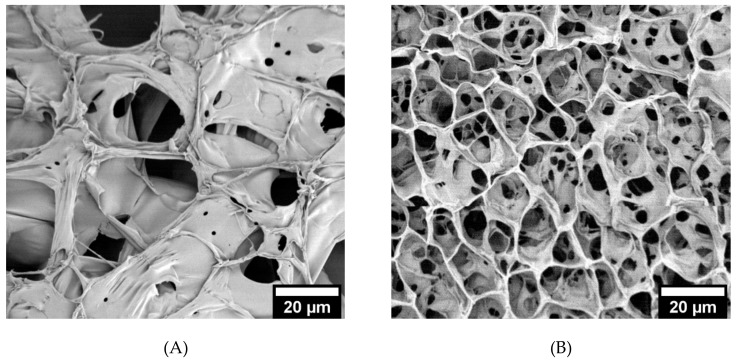

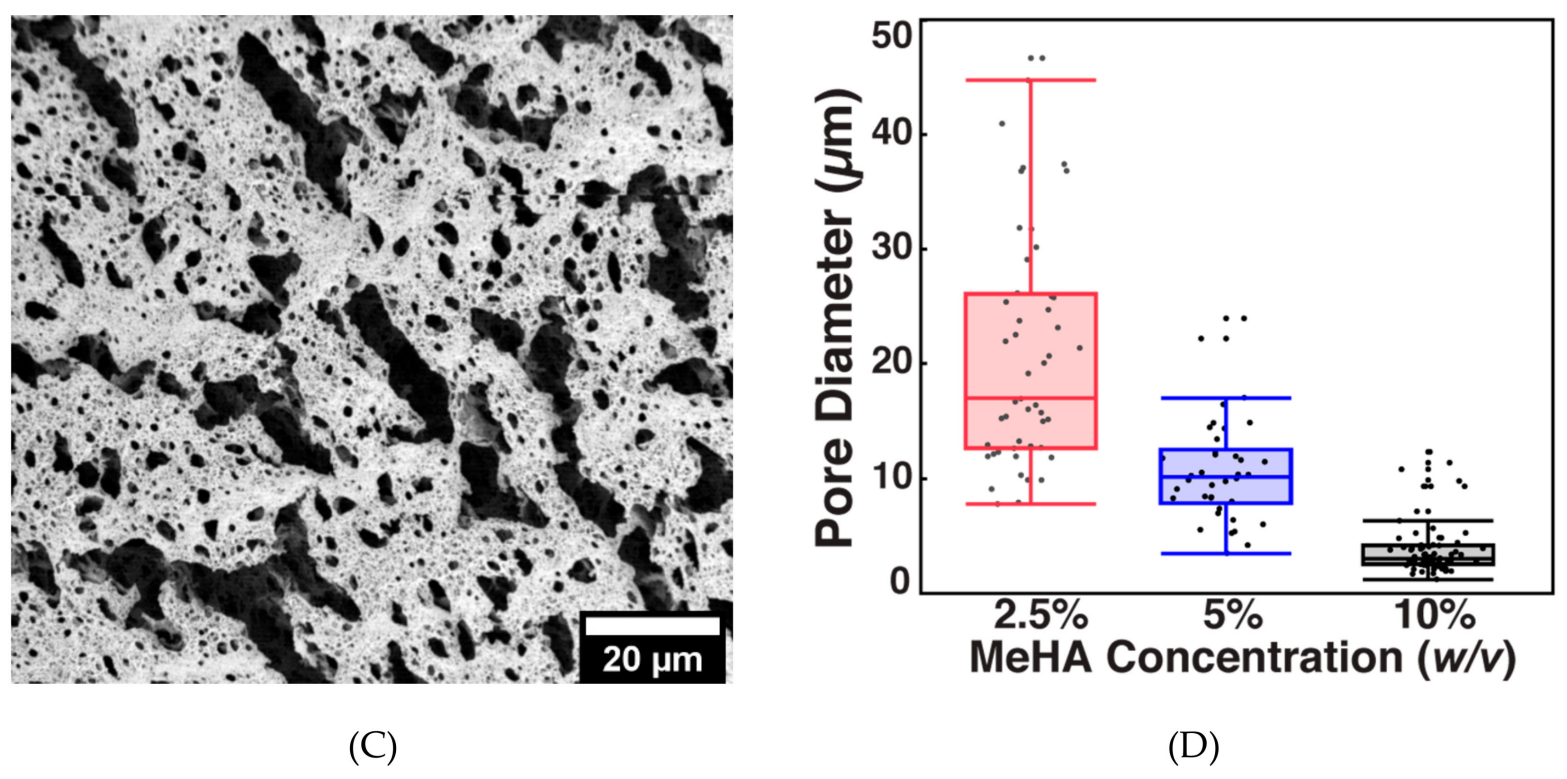
Pore size analysis of crosslinked x-MeHA hydrogels. (**A**) 2.5% *w*/*v*, (**B**) 5% *w*/*v*, and (**C**) 10% *w*/*v* hydrogels were crosslinked and lyophilized and then analyzed under SEM. (**D**) Pore diameters analyzed via ImageJ and JMP. The 5% and 10% *w*/*v* hydrogels had significantly smaller pore diameters (two-sample *t*-tests) compared to the 2.5% *w*/*v* hydrogels. Scale bar is 20 µm.

SEM micrographs (Figure 3A–C) reveal differences in crosslinking density between the hydrogel formulations. The diameters of the pores were measured in ImageJ and compared (Figure 3D). The hydrogels formed with 2.5% *w*/*v* MeHA had significantly larger pores compared to those of 5% *w*/*v* MeHA. The pore diameters of 2.5% *w*/*v* x-MeHA hydrogels measured 21.0 ± 10.2 µm, approximately two times the diameter of pores in 5% *w*/*v* x-MeHA hydrogels which measured 10.7 ± 4.5 µm. Similarly, the pore diameters of 10% *w*/*v* x-MeHA hydrogels measured 3.9 ± 2.3 µm. Thus, a trend in decreasing pore size with increasing polymer concentration was observed, as expected.

### 3.3. ReNcell VM Adhesion and Spreading on x-MeHA Hydrogels

**Figure 4:**
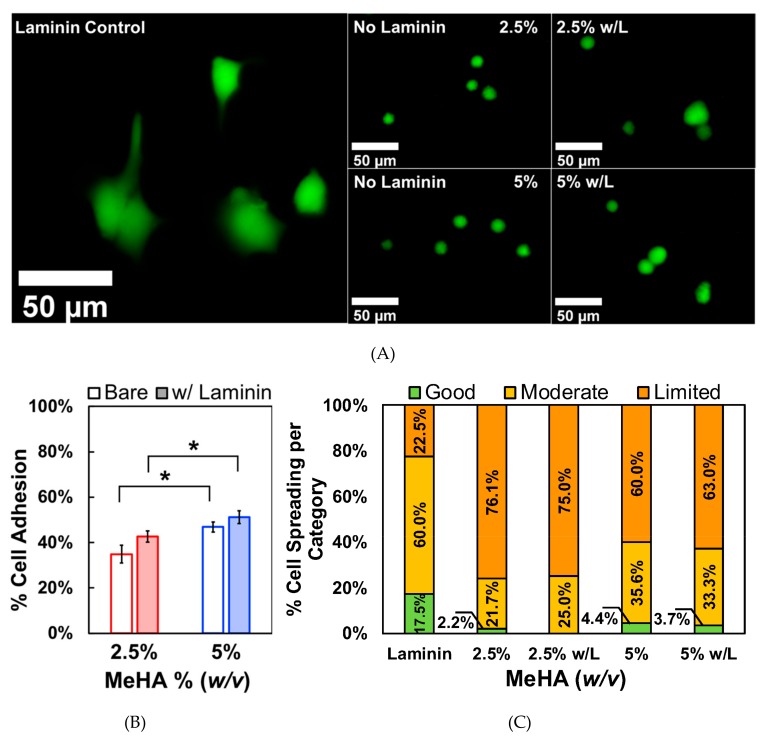
Comparison study of ReNcell VM adhesion on crosslinked x-MeHA hydrogel versus laminin-coated polystyrene. (**A**) Representative fluorescence microscopy images of cell spreading are presented. Far left laminin coating is on tissue culture plastic while the right 2 × 2 images all involve crosslinked x-MeHA hydrogels. (**B**) Significantly less cells were able to adhere to the hydrogel surfaces regardless of surface coating. Surface coating the hydrogels with laminin (w/L) improved adhesion but not significantly. In both conditions, the 5% *w*/*v* hydrogels promoted significantly, improved cell adhesion compared to 2.5% *w*/*v* hydrogels (* *p* < 0.05). Error bars are depicted as standard error. (**C**) Circularity values assigned to groups were defined as good (0–0.33), moderate (0.33–0.66), or limited (0.66–1). The percentage of cells in each category is presented for each condition (% numbers shown); w/L indicates ’hydrogels coated with laminin’ (and not a unit). Scale bar is 50 µm; *n* ≥ 3 per condition.

For the biological experiments, we selectively chose to use the 2.5% and 5% *w*/*v* x-MeHA hydrogels due to their lower modulus values that match the brain matrix (See discussion for conversion of storage modulus to Young’s modulus). When comparing the stiffness to previously published reports, the 10% *w*/*v* hydrogel resulted in a modulus range that was not ideal for neuronal differentiation [6,16]. The adhesion and spreading of ReNcell VM on the hydrogels were assessed using cells on laminin as control groups (Figure 4A). The % adhesion was calculated from a standard curve and the PrestoBlue assay [34,35,36], which measures metabolic activity. The standard curve was used to indirectly measure cell number based on resazurin conversion to resorufin. As expected, (Figure 4B), cells had significantly decreased adhesion on hydrogel surfaces compared to laminin-coated polystyrene controls. On average, only 34.9% of initial cells seeded were adhered to the 2.5% *w*/*v* hydrogels. On the 5% *w*/*v* hydrogels, an average of 46.9% of cells successfully attached to the surface, which was statistically significant compared to 2.5% *w*/*v* hydrogels. Incubating the hydrogels with laminin resulted in slightly improved cell adhesion, with 43.4% of cells attaching to the 2.5% *w*/*v* hydrogels and 53.0% attaching to the 5% *w*/*v* hydrogels. These were not statistically significant compared to uncoated hydrogels.

Cell spreading was quantified by circularity analysis (Figure 4C). Circularity is given by the following equation:

(1) $$C=\frac{4\pi A}{P^{2}}$$

where *C* is circularity, *A* is the area of the object (i.e., cell body), and *P* is the perimeter [37]. As the shape approaches that of a perfect circle, the value of *C* approaches 1. After measuring the circularity values of cells on laminin-coated and hydrogel surfaces, we categorized how well the cells spread based on circularity. We arbitrarily chose cells with circularity values between 0 to 0.33 to be assigned as the “good spreading” group. Circularity values between 0.33 to 0.66 were assigned to the “moderate spreading” group, and circularity values between 0.66 to 1 were considered to be in the “limited spreading” group. 17.5% of the cells grown on laminin-coated polystyrene had good spreading while only 2.2% and 4.4% of cells fell into this category for 2.5% and 5% *w*/*v* x-MeHA hydrogel groups, respectively (Figure 4C). 60% of the cells grown on laminin fell into the moderate spreading category. In contrast, 21.7% of cells on the 2.5% *w*/*v* hydrogels and 35.6% of cells on the 5% *w/v* hydrogels were in this category (Figure 4C, 2.5% and 5% bar graphs). The majority of the cells grown on hydrogels were in the limited adhesion category. This was 76.1% of cells on the 2.5% *w*/*v* hydrogels and 60% of cells on the 5% *w*/*v* hydrogels. 22.5% of cells grown on laminin fell into the limited spreading category. Thus, laminin coating did not significantly change the spreading characteristics of ReNcell VM on the x-MeHA hydrogel surfaces (Figure 4C, w/L bar graphs).

### 3.4. ReNcell VM Proliferation on x-MeHA Hydrogels

**Figure 5:**
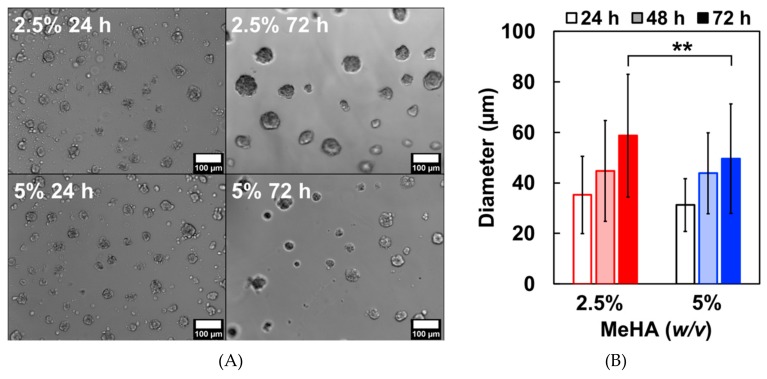
Proliferation of ReNcell VM cell clusters on laminin-coated crosslinked x-MeHA hydrogel surfaces. (**A**) The spheroids grew in size over time, as observed under live-cell microscopy. (**B**) After 72 h, spheroids on the softer 2.5% *w*/*v* x-MeHA hydrogels were significantly larger (** *p* < 0.01) compared to those on the 5% *w*/*v* x-MeHA hydrogels. Errors bars depicted as standard deviation (S.D.). Scale bar is 100 µm. *n* ≥ 3 per condition.

Next, we assessed the proliferation of ReNcell VM on the crosslinked hydrogel surfaces with laminin using live-cell microscopy. Although cells were seeded as single suspensions, isolated colonies of cell clusters were observed over time (Figure 5A). This result was expected, as NSCs are known to form neurospheres in the absence of adhesion cues [38]. The diameters (mean ± standard deviation) of these cell clusters were measured at 24, 48, and 72 h (Figure 5B). 24 h after seeding, cells formed spheroids with diameters of 35.3 ± 15.3 µm and 31.2 ± 10.5 µm on 2.5% and 5% *w*/*v* x-MeHA hydrogels, respectively. At 48 h, cells on both surfaces increased significantly in size to 44.7 ± 20.0 µm and 43.8 ± 16.1 µm on 2.5% and 5% *w*/*v* x-MeHA hydrogels, respectively. At 72 h, the spheroids on the softer 2.5% *w*/*v* x-MeHA hydrogels measured 58.7 ± 24.3 µm in diameter, which were significantly (statistically) larger than the spheroids on the 5% *w*/*v* x-MeHA hydrogels that measured 49.6 ± 21.7 µm in diameter.

### 3.5. ReNcell VM Differentiation on Crosslinked x-MeHA Hydrogels

**Figure 6:**
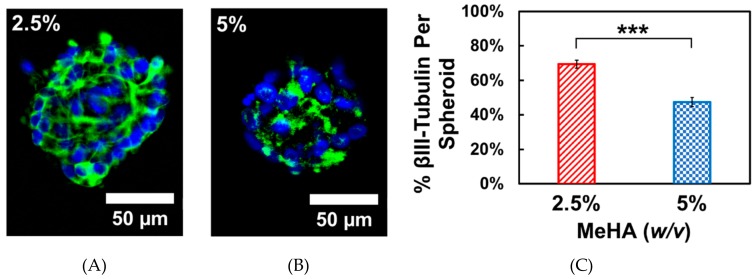
One-week Differentiation of ReNcell VM spheroids on crosslinked x-MeHA hydrogel surfaces at (**A**) 2.5% and (**B**) 5% *w*/*v* polymer concentrations. Neuronal differentiation was assessed by staining for βIII-tubulin (green). Nuclei was stained with Hoechst 33342 (blue). (**C**) Individual spheroids on the 2.5% *w*/*v* x-MeHA hydrogels had significantly increased (*** *p* < 0.001) βIII-tubulin expression compared to spheroids on 5% *w*/*v* x-MeHA hydrogels. Error bars depicted as standard error of the mean (S.E.M.). Scale bar is 50 µm; *n* ≥ 3 per condition.

While there were no significant differences in adhesion and spreading on hydrogel surfaces with laminin, we chose to use laminin for the differentiation experiments. This is because CD44 expression (HA-mediated adhesion) in NSCs is gradually decreased during differentiation [39]. After the 72-h proliferation period, cells were differentiated by withdrawing growth factors from the media. This causes spontaneous differentiation in ReNcell VM towards both neuronal and glial phenotypes [23]. We examined the neuronal differentiation capability of these cell types, which is often reported in the literature to be enhanced by softer substrates [40]. After 7 days, the cells remained as spheroids which stained positively for βIII-tubulin, a neuronal marker. The total βIII-tubulin expression was quantified by image analysis of the spheroids. The data presented in Figure 6 below was determined by taking the ratio of total βIII-tubulin positive pixels to the total area of the neurosphere in a maximum projection z-stack. In agreement with other published literature, we observed that ReNcell VM spheroids on the softer 2.5% *w*/*v* x-MeHA hydrogels expressed significantly higher levels of βIII-tubulin per spheroid compared to those on the stiffer 5% *w*/*v* x-MeHA hydrogels. The average βIII-tubulin expression per spheroid on the softer hydrogels was 69.4 ± 10.2% compared to 47.5 ± 12.8% on the stiffer 5% *w*/*v* x-MeHA hydrogels.

## 4. Discussion

In the final MeHA product, additional methacrylate peaks corresponding to the methacrylic acid by-product are observed (Figure 1). Although higher molecular weight MeHA can be further purified by ethanol precipitation [2], the oligomeric low molecular weight HA used in this study failed to precipitate, and so a phase separation method was utilized to remove the majority of the methacrylic acid byproduct. Nonetheless, MeHA synthesized from cosmetic-grade HA resulted in similar ^1^H-NMR spectra (Figure 1) compared to those previously reported using pharmaceutical-and-research-grade HA [2,41,42]. The other aspect to consider is the level of bacteria and endotoxin in the starting HA material, since most commercially-available HA is synthesized using microbial culture [43]. Interestingly, one study (from 2017) found that even the pharmaceutical- and-research-grade HA contained trace levels of endotoxin [44], which were similar to levels found in the cosmetic-grade HA reported in the product specification sheet used in this study (provided by CosChemSupply, Rancho Cucamonga, California).

Although there are other water soluble, visible-light initiated photoinitiators that have been developed [36,45], Irgacure 2959 is still considered the gold standard for biological applications [46]. In addition, by using Irgacure 2959 we could compare our biological results to those in the existing literature using similar hydrogel formulations [2,6]. We chose to use the 302 nm UV light since it is closer to the maximum absorption wavelength of Irgacure 2959 [36] and because we did not directly encapsulate cells into the x-MeHA hydrogels. When encapsulating cells using this type of crosslinking, a higher wavelength light (>365 nm) should be used to reduce the occurrence of photo-induced genotoxicity [47].

To compare the stiffness of our hydrogels (Figure 2) to those previously published in the literature for NSC differentiation [2,6,16,17], we estimated the compressive modulus (i.e., Young’s modulus) from the storage modulus. This was done by using the following equation:

(2) $$E=2G^{\prime }\left(1+\nu \right)$$

where E is the compressive modulus, G’ is the storage modulus, and ν is the Poisson’s ratio [48]. For hydrogels, the Poisson’s ratio has been often assumed to be 0.5 for calculations [16,49,50]. Using this equation, the Young’s modulus of the 2.5% and 5% *w*/*v* x-MeHA hydrogels were calculated to be 125.6 ± 14.5 Pa and 795.3 ± 60.7 Pa, respectively. The Young’s modulus of 10% *w*/*v* x-MeHA hydrogels was calculated to be 2800 ± 507.1 Pa, which was not ideal for neuronal differentiation. The 2.5% and 5% *w*/*v* x-MeHA hydrogel formulations are in the range of stiffness which have been reported to promote neuronal differentiation [4,6,16,17].

For the pore size measurements (Figure 3), it is important to note that the hydrogels were lyophilized for SEM imaging after crosslinking. Lyophilization, or freeze-drying, is a process that has been utilized to promote formation of porous networks due to ice crystallization during freezing [51,52,53,54,55]. Thus, the actual pore sizes of the hydrated hydrogel may be significantly different than those observed under SEM. This could explain why the cells were not observed to penetrate into the hydrogel, even with pore sizes are measured to be larger than the cells themselves (under SEM analysis conditions; vacuum dried); when water is added the pore walls would enlarge and make the pore sizes smaller. Solvent replacement would be an alternative strategy to measure total pore volume in the bulk hydrogel network. Absolute ethanol is commonly used, which fully fills the interstitial space and wets the sample without causing swelling of the entire network [56,57]. The total pore volume can then be calculated based on the remaining ethanol’s volume and density. We, however, did not pursue this route as it is outside the scope of this paper, since we are not trying to encapsulate cells but to establish the base culture conditions for proliferating and differentiating ReNcell VM on crosslinked x-MeHA (low molecular weight HA version; 8000–15,000 Daltons) hydrogels at the 2.5% and 5% *w*/*v* conditions.

The improved cell adhesion on the stiffer hydrogel surface may be due to the increased polymer concentration, which would theoretically increase interactions with HA receptors on the cells. HA receptors, such as CD44, have been shown to directly affect NSC adhesion [58]. Another strategy is to incorporate additional cell adhesion factors, such as ECM proteins or peptides, to enhance NSC adhesion [7,59,60]. The brain ECM is unique in the fact that many of the fibrous ECM proteins, such as collagen and fibronectin, are virtually absent compared to laminins [1]. In fact, the neurogenic zones of the endogenous NSC niche have special ECM structures called “fractones” which are rich in laminins [61]. Exogenous laminins have been reported to play important roles in promoting NSC proliferation, migration, and differentiation [62,63]. While we observed an increase in the total cell adhesion after incubating the hydrogels with laminin (Figure 4), the results were not statistically significant. This could be due to poor laminin adsorption to hydrophilic surfaces which can yield non-uniform and non-desirable conformations of the protein, resulting in reduced bioactivity [64]. Increasing the substrate stiffness has been shown to promote cell adhesion and spreading through the generation of cytoskeletal tension in several cell types [65,66,67]. Non-polymer-concentration based studies, however, are warranted in future studies where the stiffness properties of the crosslinked x-MeHA hydrogels are controlled via, for instance, light energy transfer condition modulation.

The increase in cell-cell interactions due to lack of matrix adhesion could impact the proliferation and differentiation of the NSCs. Compared to monolayer cultures, cells grown in neurospheres have some heterogeneity in terms of maturation level [68]. Thus, it may be possible that some cells in this spheroid are primed to differentiate during the proliferation phase of the study. In fact, ReNcell VM has been reported to undergo enhanced neuronal differentiation when first cultured as a neurosphere [23]. Enhanced neurogenesis is also reported in neurospheres formed from iPSC-derived neural progenitor cells (NPCs) [69] and mesenchymal stromal cells from umbilical cords [70]. The proliferative capability of the neurosphere, however, is greatly diminished as the size approaches 200–250 µm [71,72], supporting the notion that there may be an optimal size for neurosphere differentiation.

The cellular mechanism leading to stiffness-induced neuronal differentiation has been reported to be regulated by the YAP (Yes-associated protein) and TAZ (WW domain-containing transcription regulator 1 protein; Transcriptional coactivator with PDZ-binding motif) mechanosensory pathway, where inhibition of YAP by the soft substrate led to downstream activation of neurogenic genes [73]. This has also been shown in a mouse model, where neuronal differentiation was accompanied by a decrease in YAP activity [74]. On stiffer substrates, YAP/TAZ localizes to the nucleus [75] and interacts with transcription factors to maintain NSC stemness [76]. While our results agree with those in the literature reporting enhanced neuronal differentiation on soft substrates, we did not observe any extensive neurite outgrowth. This may be due to the lack of cell adhesion to the hydrogel surface, as discussed above. Cell adhesion to the surrounding ECM leads to the activation of YAP by nuclear accumulation, resulting in focal adhesion kinase-regulated cytoskeletal remodeling [77]. In NSCs, the upregulation of YAP has been shown to directly influence neurite outgrowth [78].

When we examine the native neural stem cell niche, NPCs first differentiate into immature neuroblasts, which slowly migrate as a chain of cells with minimal adhesion to the ECM [79] until they are integrated into the existing neuronal network [80,81]. Considering the role of YAP in both differentiation and neurite outgrowth, future substrate designs should incorporate a time-sequenced stiffening effect. This will allow for temporal control of YAP activity in NSCs to first promote differentiation in a soft environment inhibiting YAP, and slowly transition to a stiffer environment to increase YAP activity for neurite development and maturation. We reduced the number of physicochemical variables as much as possible to develop a general view of how ReNcell VM responds to substrate stiffness. When the cells are encapsulated in a 3D culture environment, however, then the degradability of the matrix must also be considered to promote cell migration and neurite outgrowth [82]. Since most of the softer hydrogels are made by reducing the degree of crosslinking, the differences in the degradation rates may help to explain the neurite outgrowth observed in softer hydrogels from previous studies that encapsulated NSCs [6]. Future hydrogel formulations will need to carefully introduce new physicochemical properties in a controlled manner to better elucidate the effect of each parameter on NSCs.

## 5. Conclusions

In this study, we synthesized and characterized a photochemically crosslinked hydrogel fabricated from methacrylated hyaluronic acid (MeHA) and studied the response of ReNcell VM human neural stem cells grown on the soft substrate surfaces (i.e., 125.6 ± 14.5 Pa and 795.3 ± 60.7 Pa). We used an inexpensive cosmetic-grade low molecular weight hyaluronic acid (HA) as the starting material to create the crosslinked MeHA (x-MeHA) polymeric substrates, which resulted in a similar ^1^H-NMR spectrum compared to those synthesized from more expensive, pharmaceutical-or-research-grade HA. This opens the door to the large-scale production of x-MeHA hydrogels for cost effective, high-throughput tissue engineering studies. When ReNcell VM were grown on the x-MeHA hydrogels, we observed similarities in cell adhesion, spreading, proliferation, and differentiation behavior compared to previously published literature, which studied non-human and human ESC-or-iPSC-derived NSCs in 2D and 3D microenvironments. ReNcell VM exhibited enhanced expression of βIII-tubulin on the softer hydrogel surfaces (E = 125 Pa), though no neurite outgrowth was observed. The results from this study will serve as a baseline for investigating the effects of physicochemical properties on ReNcell VM hNSCs using more complex hydrogel formulations (i.e., the inclusion of pendant peptide groups and dynamic stiffness modulation).

## Figures and Tables

**Table 1:** Degree of methacrylation calculated using the three methods described in the literature. Calculated from ^1^H-NMR spectra obtained from four different synthesis batches.

Peaks of Interest	Proton Ratio (Methacrylate to Reference)	Methacrylation % (Mean ± S.D.)	Data Boxplot
δ 6.2 ppm, δ 5.8 ppm, δ 3.0–4.2 ppm [28]	2:10	58.2 ± 19.5%	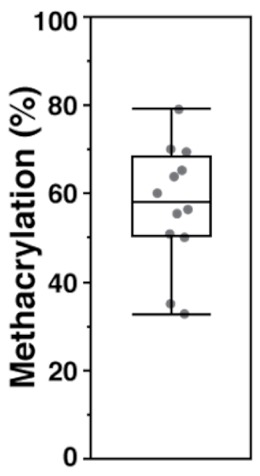
δ 6.2 ppm, δ 5.8 ppm, δ 2.0–2.1 ppm, δ 1.9–2.0 ppm [29]	5:3	59.6 ± 16.6%	
δ 6.2 ppm, δ 5.8 ppm, δ 4.2–4.8 ppm [30]	1:1	54.4 ± 4.7%	
